# Periodontal Disease Is an Independent Predictor of Intracardiac Calcification

**DOI:** 10.1155/2013/854340

**Published:** 2013-09-11

**Authors:** Gregg S. Pressman, Atif Qasim, Nitin Verma, Masami Miyamae, Kumiko Arishiro, Yasuhiro Notohara, Vitalie Crudu, Vincent M. Figueredo

**Affiliations:** ^1^Albert Einstein Medical Center and Jefferson Medical College, 5501 Old York Road, Levy, Philadelphia, PA 19141, USA; ^2^Hospital of the University of Pennsylvania, Philadelphia, PA 19104, USA; ^3^Osaka Dental University, Osaka 573-1121, Japan

## Abstract

*Background*. Periodontitis is the most common chronic inflammatory condition worldwide and is associated with incident coronary disease. *Hypothesis*. We hypothesized that periodontal disease would also be associated with cardiac calcification, a condition which shares many risk factors with atherosclerosis and is considered a marker of subclinical atherosclerosis. *Methods*. Cross-sectional study at two sites (USA and Japan) involving subjects with both clinical echocardiograms and detailed dental examinations. Semiquantitative scoring systems were used to assess severity of periodontal disease and echocardiographic calcification. *Results*. Fifty-six of 73 subjects (77%) had cardiac calcifications, and 51% had moderate to severe periodontal disease (score > 2). In unadjusted analysis, a significant relationship between periodontal score and cardiac calcification (Spearman rho = 0.4, *P* = 0.001) was noted, with increases in mean calcification score seen across increasing levels of periodontal disease. On multivariate logistic regression, adjusted for age, gender, race, glomerular filtration rate, and traditional risk factors, this association remained significant (*P* = 0.024). There was no significant interaction by study site, race, or gender. *Conclusions*. In a multiracial population, we found a significant association between the degree of periodontal disease, a chronic inflammatory condition, and cardiac calcification. Further, higher periodontal scores were associated with greater degrees of calcification.

## 1. Introduction

Periodontal disease is prevalent in the general population [1]. It results from chronic bacterial infection which destroys the attachment fibers and supporting bone that hold the teeth in place. The spectrum of periodontal disease ranges widely from gingivitis to necrotizing periodontitis, ultimately resulting in tooth loss if not treated. The chronic low-grade inflammation associated with this condition has been linked to coronary heart disease (CHD) [2–4].

Cardiac calcifications, frequently seen on echocardiography, are common in patients with CHD [5–8]. Such calcifications increase in prevalence as people age and can involve the mitral annulus, submitral apparatus, aortic root, and valvular structures. In the case of severe calcification, stenotic and regurgitant valvular disease can result. Why cardiac structures calcify is not entirely clear. Chronic kidney disease clearly plays a role, and dialysis patients frequently have severe calcification of cardiac structures [9, 10]. Many individuals without renal disease, however, also have cardiac calcifications, suggesting other underlying pathways. Cardiac calcification also shares many risk factors with atherosclerosis and is strongly associated with this chronic inflammatory disease, making it likely that the two share underlying causes. We hypothesized that periodontal disease, a common chronic inflammatory condition, would be associated with the presence and severity of cardiac calcification.

## 2. Methods

### 2.1. Study Population

The study population came from the two sites—Einstein Medical Center (Philadelphia, PA) and Osaka Dental University in Japan. The institutional review board at each institution reviewed and approved the study protocol. All individuals who had a comprehensive outpatient dental examination between April 2006 and September 2010 at either institutions were eligible for inclusion.

Echocardiography exam logs were reviewed, and subjects were included if they had undergone a clinically indicated transthoracic echocardiogram within one year (before or after) of their comprehensive periodontal evaluation. No echocardiograms were excluded because of technical limitations. Subjects were defined as having chronic kidney disease if glomerular filtration rate (GFR) was less than 60 mL/min/1.73 m^2^ (estimated using the Modification of Diet in Renal Disease or “MDRD” formula). Hypertension was defined either by report in the medical history or by a finding of systolic blood pressure >130 mm/Hg (>120 mmHg if diabetic) or diastolic >80 mmHg on multiple visits. A family history of CHD was present if at least one first-degree relative had a premature myocardial infarction. Hyperlipidemia was considered present only if part of the past medical history. Former and current smoking status was considered similar for the purposes of analysis.

Exclusion criteria for this study were the presence of rheumatic heart disease and bicuspid aortic valve. For the purpose of analysis, only one echocardiogram per subject was included; if multiple were available, the one closest in time to the dental exam was utilized. Thirty-three subjects were recruited at Einstein Medical Center and 40 at Osaka Dental University.

### 2.2. Assessment of Cardiac Calcification by Echocardiography

All echocardiograms for this study were blindly reviewed by a single echocardiographer (GSP) across the two sites. A previously published echocardiographic calcium score [11] was used to assess overall cardiac calcification in a semiquantitative fashion (Table 1). This score encompasses information on the presence and severity of calcification in the aortic valve and root, mitral valve and annulus, and submitral apparatus. Calcification is considered to be present when echo brightness of a structure exceeds that of normal valve tissue or myocardium (in the case of papillary muscle calcium). Because the pericardium is normally very echo “bright,” it is not included in the score. It is acknowledged that echocardiography cannot accurately distinguish sclerotic from calcific nodules. However, sclerosis is a precursor for calcification and may contain calcium deposits; therefore, sclerotic nodules are counted by the echocardiographic score.

### 2.3. Assessment of Periodontitis by Periodontal Scores

A comprehensive evaluation of periodontal health was performed by a dental specialist using a standardized semiquantitative scale at both facilities [12, 13]. The severity of periodontal disease was based on the depth in mm of gingival “pockets” (a pocket is the sulcus where the gum meets the tooth). The mouth was divided into 4 quadrants which were individually scored as follows: 0 = all 1–3 mm pockets (normal, healthy); 1 = any 4 mm pockets (gingivitis); 2 = any 5 mm pockets (mild periodontitis); 3  = <30% of sites have ≥6 mm pockets (moderate periodontitis); 4 = >30% of sites have ≥6 mm pockets (severe periodontitis). The average of the 4 scores (one for each quadrant) was used to produce a total mouth/patient score. Missing teeth were not included in the periodontal scoring.

### 2.4. Statistical Analysis

Crude unadjusted analysis was performed using Spearman's rank correlation coefficient. The Chi-squared test, rank sum test, and Student's *t*-test were used to compare baseline variables across those with and without cardiac calcium shown in Table 2. Subsequent adjusted analyses were performed using linear regression of the log transformed dependent variable cardiac calcification score. This was done as the natural log (calcification score + 1), as calcification score was not normally distributed, and several subjects had a 0 score. Additional analysis was performed across tertiles and quartiles of cardiac calcification score using ordinal logistic regression and yielded very similar results. Missing data from our chart review was minimal; three individuals had missing data for detailed elements of the past medical history, and 5 individuals did not have creatinine measurements. Analysis was performed in sequential multivariable models with all available data. Sequential models that were assessed were (1) age and gender adjusted; (2) age, gender, and race adjusted; (3) age, gender, race, and other cardiac risk factors which included: presence of diabetes, hyperlipidemia, hypertension, any tobacco use (past or present), family history of CHD (all from chart review), and GFR. Stata 11.0 (StataCorp LP, College Station, TX) was used for all analyses.

## 3. Results

Table 2 shows the characteristics of the study population divided into those with (*n* = 56) and without cardiac calcium (*n* = 17). Those with cardiac calcium were older, more often male, and more likely to have a history of diabetes, hyperlipidemia, and tobacco use. The periodontal score was also higher in those with cardiac calcium. Few individuals in this cohort had any chronic kidney disease.

**Figure 1:**
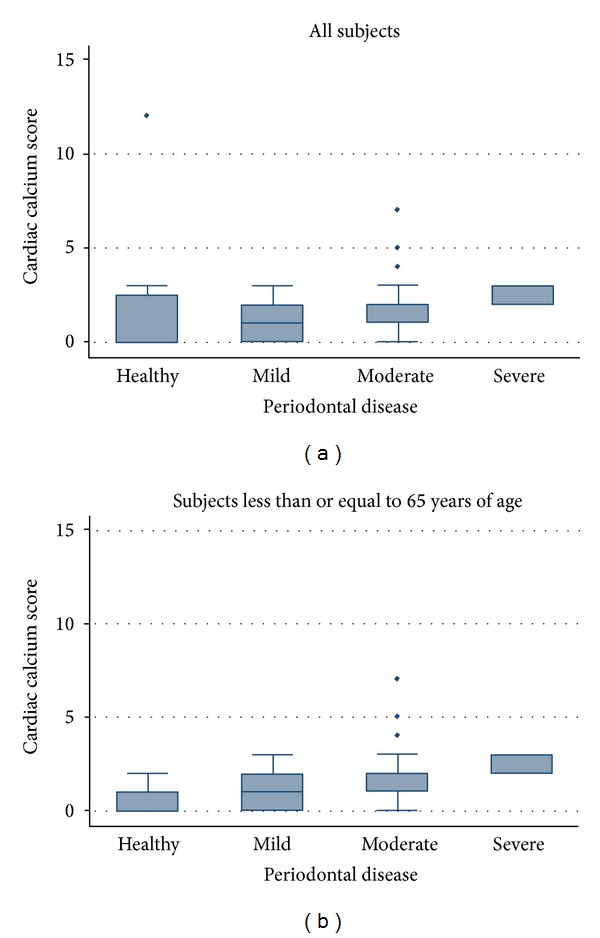
Association of periodontal disease with cardiac calcium. Box plots of cardiac calcium score across different degrees of periodontal disease. Categories for periodontal disease are based on the periodontal score (0–4 scale) as follows: healthy (0 to 1), mild (>1 to 2), moderate (>2 to 3), and severe (>3). Boxes as shown represent the interquartile ranges.

In unadjusted analysis, a significant relationship between periodontal score and cardiac calcification (Spearman rho = 0.4, *P* = 0.001) was noted, with an increase in the mean calcification score across increasing levels of periodontal disease (Figures 1(a) and 1(b)). For the entire group, there was a significant *P* value for trend, *P* = 0.001 (Figure 1(a)). If analysis was restricted to those ≤65, the trend was still significant, *P* < 0.001 (Figure 1(b) with less spread in calcification score noted in the group without periodontal disease (“healthy”). In multivariate logistic regression for the entire cohort (Table 3), after adjustment for age, gender, race, GFR, and traditional cardiac risk factors, we found a significant association between periodontal score and increasing degrees of cardiac calcification. For every one unit increase in the periodontal score, there was a 0.17 (95% CI 0.02–0.32, *P* = 0.024) unit increase in the natural log of the cardiac calcium score. We repeated the analysis across tertiles of cardiac calcium score and found similar results (data not shown). There was no significant interaction by study site (interaction *P* = 0.16), race (interaction *P* = 0.14), or gender (interaction *P* = 0.95).

## 4. Discussion

In this cross-sectional study, encompassing a diverse population across two different clinical settings, we found a significant positive correlation between the severity of periodontal disease and the degree of cardiac calcification. This is consistent with the concept that periodontal disease can be a source of chronic inflammation which can have long term consequences on cardiovascular health [4].

Calcification of cardiac structures is frequently encountered [14]. It is most commonly diagnosed through echocardiography though it is also detected by computed tomography. Previously dismissed as benign, aging related, or “degenerative” in nature, it is now understood to be an active process sharing many common risk factors with atherosclerosis [15–17]. It appears to be related to chronic inflammation, as is atherosclerosis, and has been correlated with coronary calcifications [18] and cardiovascular outcomes [5, 6, 19, 20]. Cardiac calcification is associated with chronic kidney disease [20, 21] and tends to be particularly severe in dialysis patients [10, 22]. However, it can occur in the absence of significant renal dysfunction. It is probably the case that inflammation is the key underlying mechanism while renal dysfunction accelerates the process [9, 23, 24]. 

There is no generally accepted means for quantifying cardiac calcification noted on echocardiography. Much of the existing literature focuses on calcium in the mitral annulus. However, calcific deposits elsewhere, particularly involving the aortic valve, have also been associated with atherosclerosis and cardiovascular events [14, 25, 26]. Progression of valvular calcification to a severe degree can lead to stenosis and/or regurgitation which can be clinically important. Thus, we previously developed a semiquantitative score that accounts for calcium deposits in the aortic root and valve, mitral valve and annulus, and the submitral apparatus. This score has been validated by direct comparison with calcium scoring by CT scan and has been associated with coronary calcium score [11] and severity and progression of mitral valve disease due to severe annular calcification [27].

Periodontitis is a common chronic inflammatory disease that has been linked to coronary atherosclerosis and vascular endothelial dysfunction [2, 3, 28]. A recent AHA scientific statement notes a clear association (level of evidence “A”) between periodontal disease and atherosclerotic vascular disease that is independent of known confounders [4]. Given the association between atherosclerosis and cardiac calcifications, we tested the hypothesis that periodontal disease would be associated with calcium deposits in various cardiac structures. In this research, we evaluated subjects followed in a dental clinic who had echocardiograms performed for routine clinical indications. A semiquantitative echocardiographic calcium score was applied to assess global cardiac calcification. Subjects with severe periodontal disease had significantly higher calcium scores. Further, there was a graded response, with higher periodontal scores (indicating more severe disease) associated with higher echocardiographic calcium scores. This suggests that greater degrees of chronic inflammation, as reflected by more extensive periodontal disease, contribute to greater calcification of cardiac structures. Multivariate analysis confirmed that this association was significant after controlling of age, gender, and common clinical risk factors. Restricting the analysis to those 65 and under reduced the number of outliers among the “healthy” group and produced a stronger visual association between periodontitis and echocardiographic calcium score. This suggests that periodontitis might be a more important factor in younger patients, while other factors contribute significantly to calcification in older subjects. Only 15 subjects were >65 years of age, and in this group, a definite relationship between periodontal disease and cardiac calcification was not seen; this might either be due to lack of power or because of other competing causes of cardiac calcification.

Though we did not measure coronary calcification in this study, previous investigations have found associations between periodontal disease and coronary atherosclerosis [2, 3, 28]. In addition, we and others have previously observed a correlation between the extent of cardiac calcification and the extent of coronary calcifications [11, 18] (which reflect the extent of coronary atherosclerotic disease), as well as coronary disease events [5, 6, 8]. Thus, it is likely that our subjects with cardiac calcifications had underlying coronary lesions. 

## 5. Limitations

This was a cross-sectional study, and, as such, we can only demonstrate associations and not show causation or be certain about the direction of the association. However, the intent of this work is to highlight the association of periodontal disease and cardiac calcification, a finding with clinical significance. In addition, patients were selected on the basis of having had a clinically indicated echocardiogram, and thus the study sample may not accurately represent the general population. Also the duration of the periodontal disease in the patient population is not known as it is a chronic inflammatory state. We did not include missing teeth in the periodontal score; while this may have underestimated the severity of periodontal disease, teeth may also be lost to trauma. Finally, we did not account for use of calcium channel blockers, and there is some evidence to suggest that calcium blockers may reduce the rate of calcification of cardiac structures [29]. Strengths of this investigation include (1) the multiethnic nature of the subjects, (2) blinded reading of all echocardiograms without exclusion on technical grounds, and (3) use of semiquantitative scores for measuring periodontal disease as well as cardiac calcification. The echocardiographic score we used has several strengths and limitations. The chief limitation of the echocardiographic calcium score is the inability to perfectly distinguish between calcific nodules and sclerotic nodules on echocardiographic images. However, valvular sclerosis is known to be a precursor of calcification and may even contain some calcification. Thus, the score does not try to distinguish sclerotic nodules from calcium deposits. It has some notable strengths. It accounts for the global cardiac burden of calcium rather than just focusing on the mitral annulus. It is easily applied to routine echocardiograms and does not require any additional views or sonographer time. The reading physician can quickly learn to apply the score with a minimum time added to study interpretation. Compared with other scores, there is less reliance on subjectively determined gradations in calcifications.

## 6. Conclusions

Calcification of cardiac structures is commonly observed on cardiac imaging studies. It has been associated with chronic inflammation and renal dysfunction. Once thought benign, it is now understood to be linked with atherosclerosis and can thus serve to identify patients at increased risk for future cardiovascular events. Periodontal disease has been previously associated with coronary disease. We demonstrate here that it may also be associated with markers of subclinical CHD, such as cardiac calcium. Further studies are warranted to see whether more aggressive detection and treatment of periodontal disease can have a meaningful impact on primary and secondary CHD events.

## Figures and Tables

**Table 1:** Components of the echocardiographic cardiac calcification score.

Feature	Points
(1) Posterior mitral annulus (by thirds)	None = 0 One-third calcified = 1 Two-thirds calcified = 2 Three-thirds calcified = 3
(2) Posterior mitral leaflet restriction	No = 0 Yes = 1
(3) Anterior mitral annulus involvement	No = 0 Yes = 1
(4) Anterior mitral leaflet restriction (<10 mm valve opening on the long axis view)	No = 0 Yes = 1
(5) Mitral valve calcification	No = 0 Mild = 1 >Mild = 2
(6) Subvalvular mitral apparatus calcification	No = 0 Yes = 1
(7) Aortic valve calcification	None = 0 Nodule(s) in <3 leaflets = 1 Nodules in 3 leaflets, nonrestrictive = 2 Leaflet restriction = 3
(8) Aortic root calcification	No = 0 Yes = 1

Total possible score	13

**Table 2:** Characteristics of the study population.

Variable	Cardiac calcium absent (*N* = 17)	Cardiac calcium present (*N* = 56)	*P* value
Age (±SD)	52.4 (13.3)	58.8 (11.9)	0.04
Race (%)			
Asian	41.2	60.7	0.16
Black	41.2	30.4	0.42
White	17.6	8.9	0.31
Male (%)	17.6	46.4	0.034
Diabetes (%)	12.5	18.5	0.58
Hyperlipidemia (%)	12.5	35.2	0.082
Smoking (%)	18.8	22.2	0.77
Hypertension (%)	25	29.6	0.72
Family history of CAD (%)	12.5	11.11	0.89
Chronic kidney disease (%)	0	3.7	0.44
GFR (mL/min ± SD)	106.8 (23.8)	99.7 (25.9)	0.37
Periodontal score (±SD)	1.52 (0.75)	2.31 (0.84)	01

**Table 3:** Association of periodontal score with cardiac calcification severity in multivariable models.

Model	Beta* (95% CI)	*P* value	*r* ^2^
Age, gender	0.16 (0.018–0.31)	0.028	0.174
Age, gender, race	0.20 (0.054–0.34)	0.008	0.265
Age, gender, race, risk factors^¥^	0.17 (0.02–0.32)	0.024	0.282

^*¥*^Risk factors included presence of diabetes, hypertension, hyperlipidemia, tobacco use, family history of CHD, and glomerular filtration rate in mL/min/1.73 m^2^.

*Beta represents the change in the natural log of the cardiac calcium score for every 1 unit increase in the periodontal score.
